# Prostate volume measurement by multiparametric magnetic resonance and transrectal ultrasound: comparison with surgical specimen weight

**DOI:** 10.31744/einstein_journal/2020AO4662

**Published:** 2020-01-27

**Authors:** Tatiana Martins, Thais Caldara Mussi, Ronaldo Hueb Baroni

**Affiliations:** 1 Hospital Israelita Albert Einstein São PauloSP Brazil Hospital Israelita Albert Einstein, São Paulo, SP, Brazil.; 2 Ecoar Medicina Diagnóstica Belo HorizonteMG Brazil Ecoar Medicina Diagnóstica, Belo Horizonte, MG, Brazil.

**Keywords:** Prostatectomy, Magnetic resonance imaging, Magnetic resonance spectroscopy, Ultrasonography, Prostatic diseases

## Abstract

**Objective:**

To assess accuracy of multiparametric magnetic resonance of the prostate to estimate gland volume, comparing the results with transrectal ultrasound and surgical specimen.

**Methods:**

A retrospective study of 85 patients who underwent multiparametric magnetic resonance and transrectal ultrasound (for fusion image-guided biopsy) before radical prostatectomy. Prostate measurements were obtained from magnetic resonance axial and sagittal T2-weighted images and ultrasound; the prostate volume was determined using the ellipsoid formula. The results were compared with the surgical specimen weight. Maximum interval between multiparametric magnetic resonance imaging, transrectal ultrasound, and prostatectomy was 6 months.

**Results:**

The prostate volume measured by multiparametric magnetic resonance imaging was 18-157cm^3^ (mean of 49.9cm^3^) and by transrectal ultrasound, 22-165cm^3^ (mean of 54.9cm^3^); the surgical specimen weight was 20-154g (mean of 48.6g), with no statistical differences. Based on the values obtained from imaging examinations, the prostate volume obtained was very close to the real prostatic weight, and the measures by multiparametric magnetic resonance were slightly more precise.

**Conclusion:**

Prostate volume measured by multiparametric magnetic resonance imaging and transrectal ultrasound showed similar values, and excellent agreement with real prostate weight of the surgical specimens. Prostate volume measured by magnetic resonance has been increasingly used in the clinical practice, and its value enables appropriate therapeutic planning and control of patients.

## INTRODUCTION

The estimated prostatic volume is important to help in clinical management and adequate surgical planning of patients presenting with obstructive urological symptoms related to the gland, in addition to usually being related to severity of symptoms and important in evaluating the response to the treatment prescribed.^( 1 , 2 )^ The range of therapeutic options for patients with symptoms of prostatism or prostate cancer is broad, and knowledge about the prostatic volume is important for adequate management, including for radiation therapy and brachytherapy, reducing the rate of complications, improving the results obtained, and diminishing the costs involved in treatment.^( 3 )^

The evaluation of this fact in a noninvasive manner can be indirectly estimated, based on the digital rectal examination or even contrast radiological tests, such as cystourethrography; however, with some limitations. Ultrasonography (US) has been used for many years, either suprapubic or transrectal, and the latter has greater accuracy.^( 1 - 6 )^ Currently, the most often used method is transrectal ultrasonography (TRUS), presenting with good degree of accuracy for the real prostatic weight, and has already been well established in the literature.^( 7 - 10 )^ Additionally, it is an effective method, with wide availability in the most diverse centers, and it is low cost and noninvasive.

Magnetic resonance imaging (MRI) of the prostate is increasingly more performed in clinical practice, especially in detection of suspect areas for clinically significant neoplasms, clinical follow-up of patients under active vigilance, and locoregional staging of prostate cancer. The assessment of the prostatic volume by this method has been increasingly used.^( 11 )^Despite higher costs, the MRI has the advantage of providing other pieces of information with greater accuracy than US − the main situations have been detailed above. In this way, the correct estimate of prostatic volume by this method is vital in the evaluation of these patients.

## OBJECTIVE

This study aims to compare the prostatic volume obtained by magnetic resonance of the prostate with transrectal ultrasonography, correlating both methods with the weight of the surgical specimen.

## METHODS

This is retrospective study, approved by the Research Ethics Committee (CAAE: 73587417.1.0000.0071 opinion: 2.348.860) of *Hospital Israelita Albert Einstein* . The study included patients submitted to MRI during the period from June 2013 to March 2015. All patients were posteriorly submitted to TRUS with a biopsy of the prostate by fusion image-guided image (US/MRI), followed by radical prostatectomy. The maximal interval between MRI, TRUS, and prostatectomies was 6 months. All tests were interpreted, and the measurements were obtained by radiologists with at least 5-year experience in prostate imaging. The pathologist was no aware of the values obtained by MRI and US.

All MRI were performed in 3-Tesla devices (Magnetom Trio, Siemens Healthcare, Erlangen, Germany), with the use of a surface coil and no endorectal coil, following the routine protocol of the organization, including high-resolution T2-weighted multiplanar sequences, diffusion and perfusion sequences of the prostate and seminal vesicles. The TRUS were performed on Aplio™ 500 with Smart Fusion (Toshiba Medical System Corporation, Minato, Tokyo, Japan) or LOGIC E9 with image fusion software (GE Healthcare, Little Chalfont, United Kingdom).

**Figure 1:**
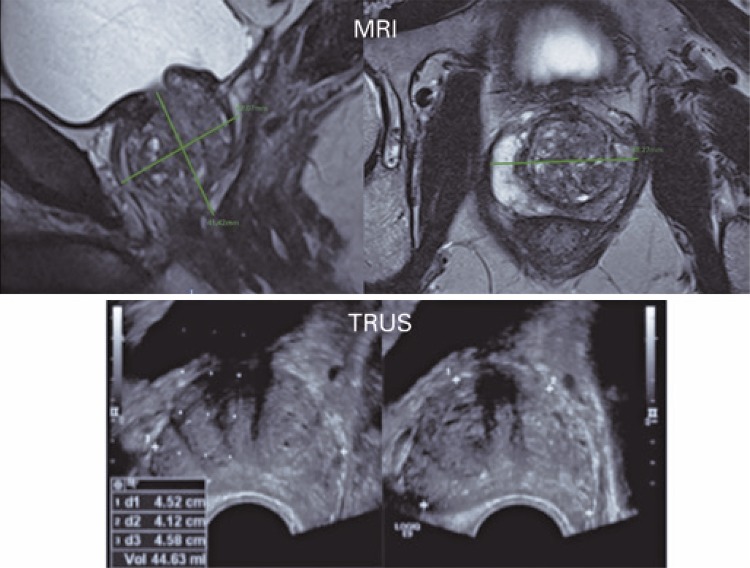
Measurements of the prostate by magnetic resonance imaging and ultrasonography on longitudinal and transverse planes MRI: magnetic resonance imaging; TRUS: transrectal ultrasonography.

The prostatic dimensions used were those documented in MRI, TRUS, and pathological examination of the surgical specimens. In MRI, the measurements were made at the work stations (Carestream, Rochester, New York, United States), based on T2-weighted axial and sagittal sequences, and the longitudinal (height) and anteroposterior diameters were obtained on the sagittal plane, and the laterolateral diameter (width) obtained on the axial plane. In TRUS, the measurements were obtained during the study, before biopsy. In both methods, the prostate volume was calculated based on the largest measurements on the longitudinal, axial, and transverse planes (ellipsoid method, calculated as follows: volume = height × width × length × 0.523) ( Figure 1 ). Values were compared with the postoperative prostatic weight (considering a 1g/mL density). All the surgical specimens were weighed after fixation with formalin, separate from the seminal vesicles.

Agreement was evaluated by the intraclass correlation coefficient, presented jointly with the confidence interval. The analyses were done with the help of packages R (1) and ir (2). Also assessed were correlation and agreement between the methods, obtained from the Bland-Altman graphs.

## RESULTS

**Figure 2:**
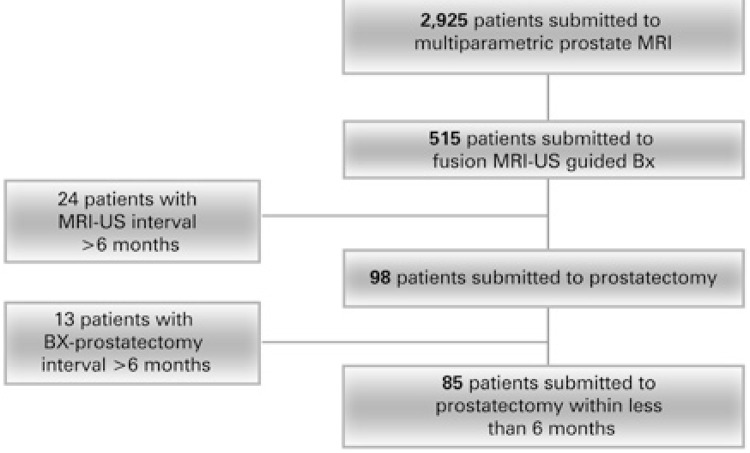
Patients included in the study MRI: magnetic resonance imaging; US: ultrasonography; Bx: biopsy.

The population studied was composed of 85 patients with prostate cancer, aged between 42 and 84 years. The prostate specific antigen (PSA) values of the sample varied from 1.4 to 26ng/mL ( Figure 2 and Table 1 ). In six cases, the measurements of the prostatic weight obtained by US were not recorded in the reports, but in all of them, the values obtained both from the MRI and surgical specimen were included, allowing a comparison between them. For this reason, they were not excluded from the study.

**Table 1:** Description of the measurements observed

	Median (1^st^ quartile-3^rd^ quartile)	Minimum value	Maximum value	n
Age, years*	63.3 (8.1)	42	84	84
Prostate volume by MRI, mL	44.0 (30.8-58.5)	18	157	84
Weight by prostatectomy, g	42.0 (32.0-52.3)	20	154	84
Volume by fusion US, mL	47.0 (32.5-59.0)	22	165	74
PSA value (ng/ml)	4.8 (3.4-6.5)	1.4	26	72

* Age described by mean and standard deviation.

MRI: magnetic resonance imaging; US: ultrasonography; PSA: prostate specific antigen.

**Figure 3:**
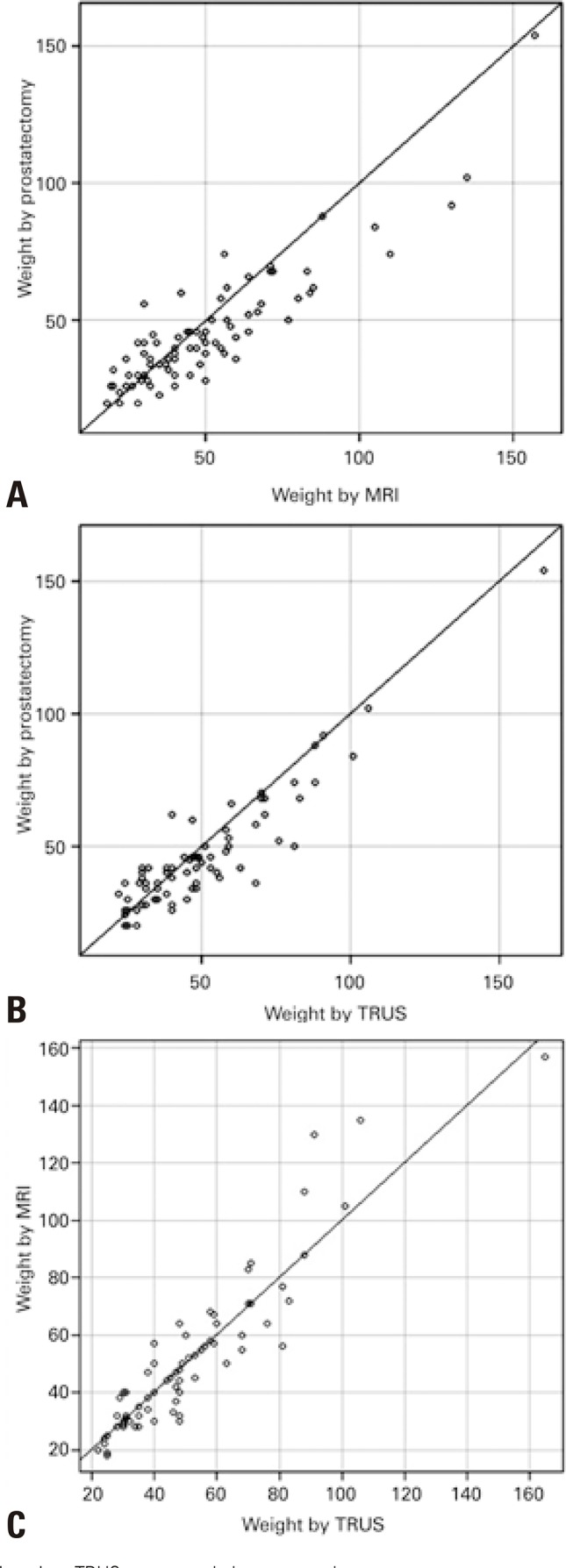
Evaluation of agreement between the prostatic weight values obtained by different methods. (A) Weights obtained by prostatectomy x magnetic resonance imaging; (B) By prostatectomy x ultrasonography; (C) By magnetic resonance imaging x ultrasonography MRI: magnetic resonance imaging; TRUS: transrectal ultrasonography.

**Figure 4:**
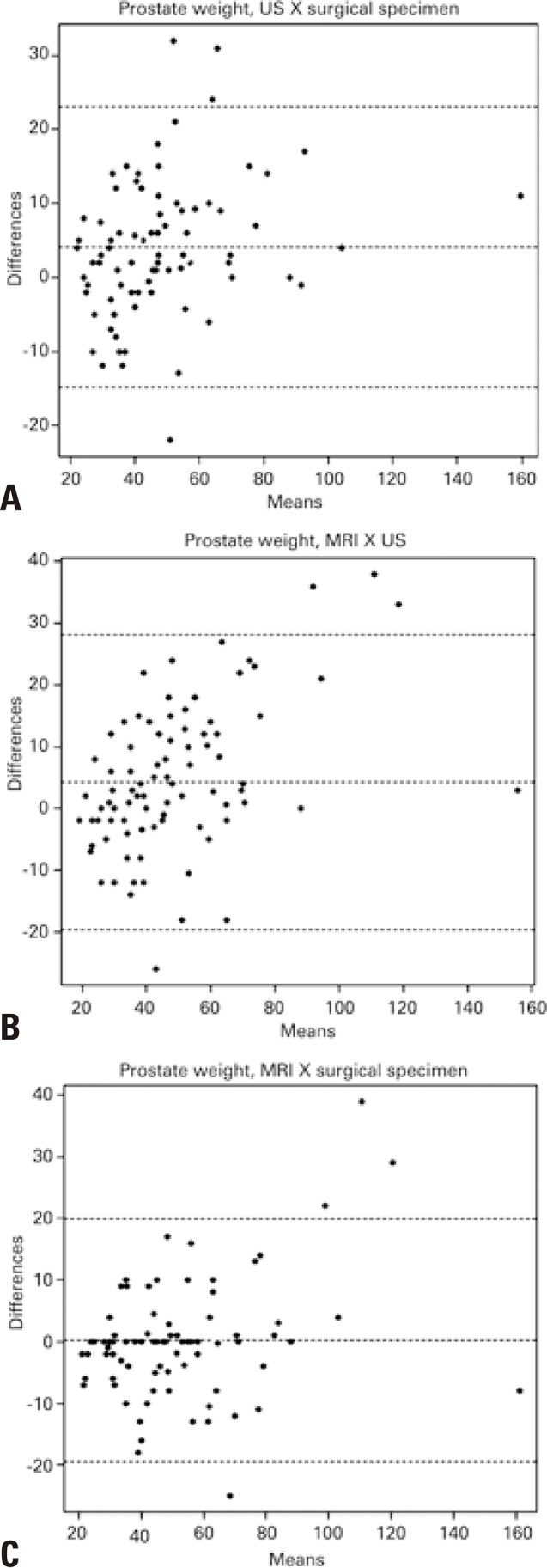
Bland-Altman graphs. (A) Correlation between prostatic weightalues obtained by ultrasonography and surgical specimen; (B) By magnetic resonance imaging and surgical specimen; (C) By magnetic resonance imaging and ultrasonography

There was no statistically significant difference between the values obtained from MRI and TRUS in assessment of prostatic weight. A high agreement between MRI and US methods was observed ( Figures 3 and 4 ). The intraclass correlation coefficient was estimated at 0.924 (95% confidence interval − 95%CI: 0.882-0.952), with a p value of 0.001, evaluating superiority at 0.85.

The intraclass correlation coefficient between the values obtained by MRI and prostatectomy specimen was estimated at 0.856 (95%CI: 0.770-0.908), p=0.445, on the test, evaluating the superiority at 0.85. The interclass correlation coefficient between the values obtained by TRUS and from the prostatectomy specimen were estimated at 0.896 (95%CI: 0.814-0.939), p=0.107 on the test, evaluating the superiority at 0.85.

## DISCUSSION

Knowledge of the prostatic volume presents with clinical and surgical implications for patients with benign or malignant modifications of the prostate. With aging, the prostate tends to increase in volume, at the expense of hyperplastic nodules of the transition zone, which can cause symptoms and hinder surgical techniques and clinical success. The correct estimate of the prostatic volume is crucial for appropriate therapeutic planning.

The volume measurement by ellipsoid calculation used in this study is obtained in a simple, quick, and precise manner, and its simplicity makes it practical for routine clinical application. Additionally, it shows good reproducibility and is used several studies to evaluate prostatic volume, as was recently shown in a published meta-analysis.^( 12 )^

The median prostatic weight based on surgical specimens obtained in our study was 42g, which is in correspondence with that found in other studies, such as by Mayer et al., which demonstrated a median weight of 47.6g, and Badani et al., of 49.9g.^( 13 , 14 )^It is possible that, in our study, this value was a little lower due to the fact that sample had many patients under 60 years of age (n=24; 28%), which could justify a lower prostatic weight due to smaller lower volume in the transition zone.^( 15 )^

This study demonstrated good agreement between the prostate volume values obtained by TRUS and surgical specimens, which is already established in literature. However, TRUS is an uncomfortable examination for patients. Moreover, it presents with diagnostic limitations, especially for the evaluation of changes in the anterior portion of the prostate and in the transition zone. In this study, we demonstrated that MRI could be used to assess prostate weight, also with a good correlation with surgical specimen weight. One advantage of our study relative to the others is the fact that we did not use the endorectal coil in routine prostate MRI, which distorts the gland anatomy and can change its correct mensuration, besides making the test more uncomfortable for the patient. The T2-weighted sequences in MRI provide better anatomic details; hence, they were used to measure the prostate in our study. Furthermore, multiplanar weighting is used in our routine protocol. Since the MRI is increasingly used in clinical practice, the measurement and consequent definition of management related to the prostatic volume can then be defined based on this method.

Ours is one of the few studies in which all patients were evaluated by MRI and TRUS within a short interval,^( 2 , 16 )^reducing the possibility of a significant progressive modification of the prostatic volume, and confirming what had been previously demonstrated as to the good correlation of measurements obtained by MRI and TRUS. A matter that could be raised would be that the interval between the MRI and prostatectomy in some cases was greater than between US and surgery, which would disfavor the volume evaluation by MRI. However, considering the similar results obtained by the two imaging methods, this possibility becomes unlikely.

Some authors considered equal values for prostatic volume and weight, since the density of the prostate is approximately 1.0g/mL.^( 17 - 19 )^ In this study, the density of 1.0 was emplyed, which was also used by Rodriguez et al.,^( 17 )^ among other authors. The value of a density of 1.05 is also widely used, and more seldom, the coefficient 1.1, as Tewari et al.,^( 16 )^published. Yet, it would be possible to extrapolate, without scientific evidence, the fact that a lower coefficient would be ideal for an adequate comparison with heavy specimens after fixation with formalin, as in this study, since they would lose water, thus reducing their weight a little.

One of the problems in the literature related to the comparison of the volumes obtained by tests with the weight recorded after prostatectomy is the fact that formalin fixation could promote weight loss, decreasing the true *in vivo* weight.^( 20 )^On the other hand, the true volume of the prostate could be overestimated by the fact that, during resection of the specimen, the prostate is usually not completely isolated from the seminal vesicles and even from the periprostatic fat. Considering there may be fragments of these structures influencing in surgical specimen weight and affecting an adequate correspondence between the volumes obtained by the tests and by the specimen. Additionally, how surgical specimens are obtained is rarely described in studies, thus limiting the precise comparison between results. In this way, even with a very accurate method, there can be limitations that hinder its adequate validation for the exact calculation of the prostatic volume. In this study, the weight of the specimens was obtained after fixation with formalin, and the prostate was previously isolated from the seminal vesicles, reducing the bias related to overlapping of volume of these structures.

One of the limitations of the study was the fact that the prostate measurements were made by different examiners, in MRI, US, as well as in pathological evaluation. Another limitation is that this is a retrospective study, based on reports available.

## CONCLUSION

The prostatic volume obtained by magnetic resonance and transrectal ultrasonography showed a good correlation with the prostatic weight obtained from the surgical specimens. Thus, the evaluation of this data based on magnetic resonance imaging, a method increasingly used in clinical practice, allows adequate therapeutic planning and clinical control of patients.
