# Supplementary material for: Prostate volume measurement by multiparametric magnetic resonance and transrectal ultrasound: comparison with surgical specimen weight
Source: Einstein (Sao Paulo). 2020 Jan 27;18:eAO4662. doi: 10.31744/einstein_journal/2020AO4662 (PMC6986883; doi:10.31744/einstein_journal/2020AO4662)
Supplement: Translation

# Translation: Avaliação do volume prostático obtido por ressonância magnética multiparamétrica da próstata e ultrassonografia transretal comparado ao peso da peça cirúrgica

**Authors:** Tatiana Martins, Thais Caldara Mussi, Ronaldo Hueb Baroni

**Affiliations:** 1 Brasil Hospital Israelita Albert Einstein, São Paulo, SP, Brasil.; 2 Brasil Ecoar Medicina Diagnóstica, Belo Horizonte, MG, Brasil.

**Keywords:** Prostatectomia, Imagem por ressonância magnética, Espectroscopia de ressonância magnética, Ultrassonografia, Doenças prostáticas

## Abstract

**Objetivo:**

Avaliar a acurácia da ressonância magnética multiparamétrica da próstata para estimativa do volume da glândula, comparando seus resultados com a ultrassonografia transretal e correlacionando com o volume obtido da peça cirúrgica.

**Métodos:**

Estudo retrospectivo incluindo 85 pacientes submetidos à ressonância magnética e, posteriormente, à ultrassonografia transretal (para orientação de biópsia com fusão de imagens) e, a seguir, à prostatectomia radical. As dimensões prostáticas foram obtidas na ressonância a partir das imagens nos planos axial e sagital em sequências ponderadas em T2 e, assim como na ultrassonografia, o volume foi calculado a partir do método da elipsoide. Os valores foram comparados com o peso prostático pós-cirúrgico. O intervalo máximo entre a ressonância e ultrassonografia e prostatectomia foi de 6 meses.

**Resultados:**

O volume prostático obtido por ressonância magnética foi de 18 a 157cm^3^(média de 49,9cm^3^); pela ultrassonografia transretal, foi de 22 a 165cm^3^(média de 54,9cm^3^); e o peso da peça cirúrgica foi de 20 a 154g (média de 48,6g), sem diferenças estatísticas. A partir do valor obtido por esses métodos de imagem, provou-se que o volume prostático obtido aproximou-se bastante do peso real da próstata, com discreta maior precisão das medidas obtidas por ressonância magnética multiparamétrica.

**Conclusão:**

As medidas do volume prostático adquiridas pela ressonância magnética e pela ultrassonografia transretal são semelhantes entre si, com excelente concordância com os pesos reais das próstatas obtidos das peças cirúrgicas. A avaliação desse dado, a partir da ressonância, método cada vez mais utilizado na prática clínica, permite o adequado planejamento terapêutico e o controle dos pacientes.

## INTRODUÇÃO

A estimativa do volume prostático é importante para auxiliar no manejo clínico e para a adequada programação cirúrgica dos pacientes que apresentam sintomas urológicos obstrutivos relacionados à glândula, além de usualmente estar relacionada à gravidade dos sintomas e ser importante na avaliação da resposta ao tratamento empregado.^1 , 2^ O leque de opções terapêuticas para pacientes com sintomas de prostatismo ou câncer de próstata é amplo, sendo o conhecimento do volume prostático importante para o manejo adequado, inclusive para radioterapia e braquiterapia, reduzindo a taxa de complicações, melhorando os resultados obtidos e diminuindo os custos envolvidos no tratamento.^3^

A avaliação desse dado de forma não invasiva pode ser estimada indiretamente a partir do toque retal ou mesmo de exames radiológicos contrastados, como cistouretrografia, no entanto, com limitações. Há muitos anos, uma ferramenta bastante utilizada tem sido a ultrassonografia (US), seja por via suprapúbica ou transretal – esta, com maior acurácia.^1 - 6^ O método mais empregado, no momento, é a ultrassonografia transretal (USTR), apresentando boa acurácia com o peso prostático real, o que já está bem estabelecido na literatura.^7 - 10^ Além disso, é um método eficaz, com ampla disponibilidade nos mais diversos centros, barato e não invasivo.

A ressonância magnética (RM) da próstata está cada vez mais difundida na prática clínica, sobretudo na detecção de áreas suspeitas para neoplasia clinicamente significante, acompanhamento clínico de pacientes em vigilância ativa e estadiamento locorregional do câncer de próstata. A avaliação do volume prostático por esse método tem sido cada vez mais utilizada.^11^ Apesar do maior custo, a RM apresenta como vantagem fornecer outras informações com maior acurácia que a US − as principais acima pormenorizadas. Dessa forma, a estimativa correta do volume prostático por esse método é crucial na avaliação desses pacientes.

## OBJETIVO

Este estudo visa comparar o volume prostático obtido por ressonância magnética da próstata com a ultrassonografia transretal, correlacionando ambos os métodos com o peso obtido da peça cirúrgica.

## MÉTODOS

Estudo retrospectivo, aprovado pelo Comitê de Ética em Pesquisa. (CAAE: 73587417.1.0000.0071 parecer: 2.348.860) do Hospital Israelita Albert Einstein. Foram incluídos pacientes submetidos à RM durante o período de junho de 2013 e março de 2015. Todos os pacientes foram posteriormente submetidos à USTR com biópsia da próstata pelo método de fusão de imagens US/RM e à prostatectomia radical. O intervalo máximo entre RM, USTR e prostatectomias foi de 6 meses. Todos os exames foram interpretados, e as medidas foram obtidas por radiologistas com experiência de, ao menos, 5 anos em imagem de próstata. O patologista não tinha conhecimento sobre dos valores obtidos por RM e US.

Todas as RM foram realizadas em aparelhos de 3 Tesla (Magnetom Trio, Siemens Healthcare, Erlangen, Alemanha), com a utilização de bobina de superfície e sem bobina endorretal, com o protocolo rotina da instituição, incluindo sequências multiplanares de alta resolução ponderadas em T2, sequências de difusão e de perfusão da próstata, e englobando a próstata e as vesículas seminais. Os exames de USTR foram realizados em um dos aparelhos: Aplio™ 500 with Smart Fusion (Toshiba Medical System Corporation, Minato, Tóquio, Japão) ou LOGIC E9 com software de fusão das imagens (GE Healthcare, Little Chalfont, Reino Unido).

As dimensões prostáticas utilizadas foram aquelas que constavam nos laudos dos pacientes de RM, USTR e do anatomopatológico da peça cirúrgica. Na RM, as medidas foram realizadas na estação de trabalho (Carestream, Rochester, Nova York, Estados Unidos), a partir das imagens nos planos axial e sagital em sequências ponderadas em T2, sendo os diâmetros longitudinal (altura) e anteroposterior obtidos no plano sagital e o diâmetro laterolateral (largura) obtido no plano axial. Na USTR, as medidas foram obtidas durante a realização do estudo, previamente à biópsia. Nos dois métodos, o volume da próstata foi calculado a partir das maiores medidas nos planos longitudinal, axial e transverso (método da elipsoide, sendo calculado da seguinte forma: volume = altura × largura × comprimento × 0,523) ( Figura 1 ). Os valores foram comparados com o peso prostático pós-cirúrgico (considerando densidade de 1g/mL). Todas as peças cirúrgicas foram pesadas após fixação com formol, separadamente das vesículas seminais.

**Figura 1:** Medidas da próstata por ressonância magnética e ultrassonografia nos planos longitudinal e transverso RM: ressonância magnética; USTR: ultrassonografia transretal.

A concordância foi avaliada pelo coeficiente de correlação intraclasse, apresentado juntamente do intervalo de confiança. As análises foram realizadas com o auxílio dos pacotes R (1) e ir (2). Foram avaliadas ainda medidas de correlação e concordância entre os métodos, obtidas a partir dos gráficos de Bland-Altman.

## RESULTADOS

A população estudada foi composta por 85 pacientes com câncer de próstata, com idade entre 42 e 84 anos. Os valores de antígeno prostático específico (PSA) da amostra variaram entre 1,4 e 26ng/mL ( Figura 2 e Tabela 1 ). Em seis casos, as medidas do peso prostático obtidos por US não constavam nos relatórios, mas em todos eles os valores obtidos tanto por RM quanto da peça cirúrgica foram incluídos, permitindo a comparação entre eles. Por esse motivo, não foram excluídos do estudo.

**Figura 2:** Pacientes incluídos no estudo RM: ressonância magnética; US: ultrassonografia; BX: biópsia.

**Tabela 1:** Descrição das medidas observadas

	Mediana (1º quartil-3º quartil)	Valor mínimo	Valor máximo	n
Idade, anos*	63,3 (8,1)	42	84	84
Volume da próstata por RM, mL	44,0 (30,8-58,5)	18	157	84
Peso por prostatectomia, g	42,0 (32,0-52,3)	20	154	84
Volume por US fusão, mL	47,0 (32,5-59,0)	22	165	74
Valor do PSA (ng/ml)	4,8 (3,4-6,5)	1.4	26	72

* Idade descrita por média e desvio padrão.

RM: ressonância magnética; US: ultrassonografia; PSA: antígeno prostático específico.

Não houve diferença estatisticamente significante entre os valores obtidos por RM e USTR na avaliação do peso prostático. Observamos alta concordância entre os métodos RM e US ( Figuras 3 e 4 ). O coeficiente de correlação intraclasse foi estimado em 0,924 (intervalo de confiança de 95% − IC95%: 0,882-0,952), com valor de p de 0,001, avaliando superioridade a 0,85.

**Figura 3:** Avaliação da concordância entre os valores dos pesos prostáticos obtidos por diferentes métodos. (A) Pesos obtidos por prostatectomia x ressonância magnética; (B) Por prostatectomia x ultrassonografia; (C) Por ressonância magnética x ultrassonografia RM: ressonância magnética; US: ultrassonografia.

**Figura 4:** Gráficos de Bland-Altman. (A) Correlação entre os valores dos pesos prostáticos obtidos por ultrassonografia e peça cirúrgica; (B) Por ressonância magnética e peça cirúrgica; (C) Por ressonância magnética e ultrassonografia RM: ressonância magnética; US: ultrassonografia.

O coeficiente de correlação intraclasse entre os valores obtidos por RM e da peça da prostatectomia foi estimado em 0,856 (IC95%: 0,770-0,908), com valor de p de 0,445 no teste, avaliando superioridade a 0,85. O coeficiente de correlação intraclasse entre os valores obtidos por USTR e da peça da prostatectomia foi estimado em 0,896 (IC95%: 0,814-0,939), com valor de p de 0,107 no teste, avaliando superioridade a 0,85.

## DISCUSSÃO

O conhecimento do volume prostático apresenta implicações clínicas e cirúrgicas para pacientes com alterações benignas ou malignas da próstata. Com o envelhecimento, a próstata tende a aumentar de volume, à custa de nódulos de hiperplasia da zona de transição, o que pode causar sintomas e também dificultar as técnicas cirúrgicas e o sucesso clínico. A estimativa correta do volume prostático é crucial para o adequado planejamento terapêutico.

A medida do volume pela técnica do elipsoide, utilizada neste estudo, é obtida de forma simples, rápida e precisa, e sua simplicidade a torna prática para a aplicação clínica de rotina. Além disso, apresenta boa reprodutibilidade, sendo utilizada em grande parte dos estudos para avaliação do volume prostático, como demonstrado em recente metanálise publicada.^12^

A mediana dos pesos prostáticos baseados nas peças cirúrgicas obtida em nosso estudo foi de 42g, o que está em correspondência com o encontrado em outros trabalhos, como de Mayer et al., que demostraram mediana de 47,6g, e Badani et al., que demostraram peso de 49,9g.^13 , 14^ É possível que, em nosso estudo, este valor tenha sido um pouco menor pelo fato de a casuística apresentar muitos pacientes com menos de 60 anos (n=24; 28%), o que poderia justificar o menor peso prostático decorrente do menor volume da zona de transição.^15^

O presente estudo demonstrou boa concordância entre os valores obtidos de volume prostático pela USTR e as peças cirúrgicas, o que já é estabelecido na literatura, entretanto, a USTR é um exame desconfortável para o paciente. Além disso, apresenta limitações diagnósticas, sobretudo para avaliação de alterações na porção anterior da próstata e na zona de transição. Neste estudo, demonstramos que a RM pode ser utilizada para avaliação do peso prostático, também com boa correlação com o peso da peça cirúrgica. Uma vantagem do nosso estudo em relação aos outros está no fato de não utilizarmos bobina endorretal na rotina da RM de próstata, o que distorce a anatomia glandular e pode alterar sua correta mensuração, além de tornar o exame mais desconfortável para o paciente. Pela RM, as sequências ponderadas em T2 fornecem melhor detalhamento anatômico e, por isso, foram utilizadas para mensuração da próstata em nosso estudo. Ainda, é a ponderação multiplanar utilizada em nosso protocolo de rotina. Visto que a RM é cada vez mais utilizada na prática clínica, a mensuração e a consequente definição de conduta relacionada ao volume prostático podem, então, ser definidas a partir deste método.

O nosso é um dos poucos estudos em que todos os pacientes foram avaliados pela RM e pela USTR em curto intervalo de tempo,^2 , 16^ reduzindo a possibilidade de significativa alteração evolutiva do volume prostático e comprovando o demonstrado previamente, sobre a boa correlação das medidas obtidas por RM e USTR. Um questionamento que poderia ser levantado seria que o intervalo entre a RM e a prostatectomia foi maior em alguns casos que o entre a US e a cirurgia, o que desfavoreceria a avaliação do volume pela RM, porém, visto a semelhança entre os resultados obtidos pelos dois métodos de imagem, essa possibilidade torna-se pouco provável.

Alguns autores consideram valores iguais para volume e peso prostático, pois a densidade da próstata é aproximadamente de 1,0g/mL.^17 - 19^ Nesse estudo, considerou-se a densidade de 1,0, também utilizada por Rodriguez et al.,^17^ dentre outros autores. O valor da densidade de 1,05 é também amplamente utilizado e, mais raramente, emprega-se o coeficiente 1,1, como publicado por Tewari et al.^16^ Seria possível, ainda, extrapolar, sem evidência científica, o fato de que um coeficiente menor seria o ideal para adequada comparação com peças pesadas após fixação com formol, como nesse estudo, pois elas perderiam água, o que poderia reduzir um pouco seu peso.

Um dos problemas na literatura relacionados à comparação dos volumes obtidos por exames com o peso registrado após prostatectomia encontra-se no fato de que a fixação por formol poderia promover perda de peso, diminuindo o peso real *in vivo.* ^20^ Por outro lado, o volume real da próstata poderia ser superestimado, pelo fato de que, durante a ressecção da peça, dificilmente a próstata é completamente isolada das vesículas seminais e mesmo da gordura periprostática, podendo haver fragmentos destas, influenciando no peso da peça cirúrgica e afetando a adequada correspondência entre os volumes obtidos pelos exames e pela peça. Além disso, a forma como o peso da peça cirúrgica é obtido raramente é descrita nos estudos, limitando a precisa comparação entre os resultados. Desta forma, por mais acurado que seja o método, podem existir limitações que dificultem sua adequada validação para cálculo exato do volume prostático. Nesse estudo, os pesos das peças foram obtidos após fixação com formol, sendo que a próstata fora isolada previamente das vesículas seminais, reduzindo o viés relacionado à sobreposição dos volumes dessas estruturas.

Uma das limitações do estudo encontra-se no fato de as medidas da próstata terem sido realizadas por diferentes examinadores, tanto na RM e US, quanto na avaliação anatomopatológica. Outra limitação é ser um estudo retrospectivo, baseado nos relatórios disponibilizados.

## CONCLUSÃO

O volume prostático obtido por ressonância magnética e ultrassonografia transretal da próstata apresenta boa correlação com o peso prostático obtido das peças cirúrgicas. Desta forma, a avaliação deste dado a partir da ressonância magnética, método cada vez mais utilizado na prática clínica, permite adequado planejamento terapêutico e controle clínico dos pacientes.
